# Supplementary figures and images for: An integrated augmented reality surgical navigation platform using multi-modality imaging for guidance
Source: PLoS One. 2021 Apr 30;16(4):e0250558. doi: 10.1371/journal.pone.0250558 (PMC8087077; doi:10.1371/journal.pone.0250558)

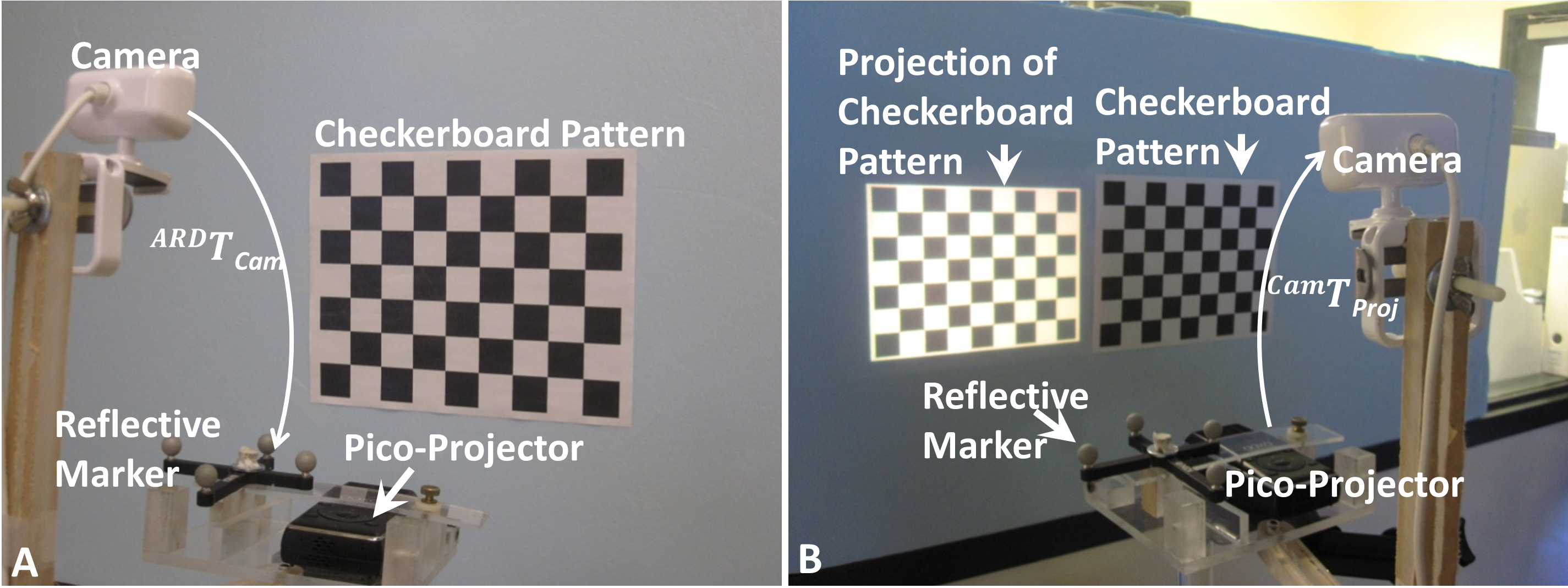

Supplement: S1 Fig — (TIF) [file pone.0250558.s001.tif]
