# Supplementary material for: An integrated augmented reality surgical navigation platform using multi-modality imaging for guidance
Source: PLoS One. 2021 Apr 30;16(4):e0250558. doi: 10.1371/journal.pone.0250558 (PMC8087077; doi:10.1371/journal.pone.0250558)
Supplement: S1 File — (DOCX) [file pone.0250558.s003.docx]

**An integrated augmented reality surgical navigation platform using multi-modality imaging for guidance**

Harley H.L. Chan PhD^1*^; Stephan K. Haerle^2^, MD; Michael J. Daly^1^ PhD; Jinzi Zheng^1^ PhD; MBA; Lauren Philp^5,6^ MD, MSc; Marco Ferrari^1,3,7^ MD; Catriona M. Douglas^1,3,4^ MBChB, MD; Jonathan C. Irish^1,3,4^ MD,MSc

^1^TECHNA Institute, University Health Network, Toronto, ON, Canada

^2^Center for Head and Neck Surgical Oncology and Reconstructive Surgery, Hirslanden Clinic, Lucerne, CH-6006, Switzerland

^3^Department of Otolaryngology-Head and Neck Surgery, University of Toronto, Toronto, ON, Canada

^4^Department of Surgical Oncology, Princess Margaret Cancer Centre, University Health Network, Toronto, ON, Canada

^5^Institute of Medical Science, University of Toronto, Toronto, ON, Canada

^6^Department of Obstetrics and Gynecology, University of Toronto, ON, Canada

^7^Unit of Otorhinolaryngology – Head and Neck Surgery, University of Brescia, Brescia, Italy

*Corresponding Author: Harley H.L. Chan, PhD, 101 College Street, 7^th^ floor, Princess Margaret Cancer Research Tower, Toronto, Ontario, M5G 1L7. Phone: (416) 581-7523 E-mail: [Harley.Chan@rmp.uhn.ca](mailto:Harley.Chan@rmp.uhn.ca)

**Spatial calibration of pico-projector and tracking tool**

The objective of pico-projector and tracker tool calibration is to maintain accuracy of projection image correctly overlay computer-generated information onto surgical surface even the system maneuvers to different location. The result of calibration is to define the tracking tool and projector spatial parameters including translational and rotational attribute. This calibration involves two steps: 1) Camera and tool calibration and 2) Camera and projector calibration. S1 Fig describes both step of calibration procedure that establishes the spatial relationship between the camera, projector and tracker tool.

**Camera and tracking tool calibration**

Camera and tracking tool calibration step is to define spatial relationship between centre of camera and origin of tracking tool coordinate in term of homogeneous transformation matrix that consist of 3x3 rotational matrix $\mathbf{R}$ and 3x1 translational vector $\mathbf{t}$. The definition of rotation parameter could also be represent as Euler angles $(R_{x},R_{y},R_{z})$. The calibration process commence with photographing a known dimension of planer checkerboard pattern from various prospective using tracked camera, the correspondent tracking tool position and orientation are recorded to compute spatial transformation. Via optimization process, the grid dimension of checkerboard from each photograph compares to real grid dimension to determine intrinsic and extrinsic parameter of camera. The optimization process employs open-source camera calibration toolbox[[1](#_ENREF_1)] for Matlab (R2010b, MathWorks Inc., Natick, Massachusetts). The calibration uses pinhole camera model to describe relationship between 3D space and projection onto the 2D coordinate image plane. Intrinsic parameters provide characteristic of the camera including focal length, principal point, skew coefficient and lens distortions. The detail description of the model and methodology of camera calibration refers to the literatures[[2-4](#_ENREF_2)]. The checkerboard pattern used in the calibration has a dimension of 30 mm x 30 mm with 9 x 7 grids array fabricated from laser printer in resolution 600 dpi on A4 paper. The extrinsic parameter represents rigid transformation of each prospective image from world coordinate (in checkerboard space) to camera. Combining both intrinsic $\mathbf{A}$ and extrinsic $[\mathbf{R t}]$ parameters, the 3D space ${\mathbf{M}\boldsymbol{=[}X,Y,Z\boldsymbol{,}1\boldsymbol{]}}^{T}$ can map to 2D camera image ${\mathbf{m}=[u,v,1]}^{T}$ presenting by

$s\mathbf{m}=\mathbf{A}\left[ \mathbf{R} \mathbf{t} \right]\mathbf{M}$ where,$\mathbf{A}=\left[ \begin{matrix} \alpha& c & u_{0} \\ 0 & \beta& v_{0} \\ 0 & 0 & 1 \end{matrix} \right]$

$s$ is an arbitrary scale factor. $\mathbf{A}$ is the camera intrinsic matrix contain $(u_{0,}v_{0})$ principal point, $\alpha$ and $\beta$ the scale factors, and $c$ skewness of the two image axes.

**Camera and pico-projector calibration**

The objective of the camera and pico-projector calibration procedure is to determine the transformation matrix between camera and projector ${{}^{Cam}T}_{Proj}$. The projector model as inverse pinhole camera that mapping 2D image to 3D rays, the fundamental principle similar to camera calibration that previously described. The intrinsic and extrinsic parameters compute by mapping 2D image data point to correspond 3D data points using nonlinear minimization technique such as Levenberg-Marquardt method. The 3D data points acquire by projecting 2D image of known dimension checkerboard pattern on the planer surface in which a secondary real checkerboard pattern co-exist on the same surface defining the plan definition. The camera on AR device captures images of both projection and real checkboard pattern (S1 Figure B) from various prospective during calibration procedure. Acquired image set import to Projector-Camera calibration toolbox[[5](#_ENREF_5)] to compute transformation of camera and projector ${{}^{Cam}T}_{Proj}$. Combining the result from 1) camera and tracker tool calibration and 2) camera and pico-projector calibration, the resulting transformation matrix from AR device (ARD) to camera is ${{}^{ARD}T}_{Proj}={{}^{ARD}T}_{Cam}*{{}^{Cam}T}_{Proj}$

S1 Fig: (A) First step of camera-projector calibration for the mobile hand-held device. This step is to define the spatial relationship between the camera and reflective marker. During the calibration procedure, the AR device re-locates at different positions for the camera to capture an image of checkerboard pattern at each location. Simultaneously, the corresponding pose and orientations of the reflective marker are recorded. (B) Second step of the camera-projector calibration for the mobile hand-held device. During the calibration procedure, the AR device re-locates at different positions for the camera to capture an image including both the projection image of the checkerboard pattern and the real checkerboard pattern. The aim of this procedure is to define the spatial relationship between camera and pico-projector. Combining this with the results from first procedure, the spatial relationship can determined and represented by a homogeneous transformation matrix.

**References:**

1. Bouguet J-Y (2004) Camera calibration toolbox for matlab.

2. Heikkila J, Silvén O. A four-step camera calibration procedure with implicit image correction; 1997. IEEE. pp. 1106-1112.

3. Tsai RY (1992) A versatile camera calibration technique for high-accuracy 3D machine vision metrology using off-the-shelf TV cameras and lenses. In: Lawrence BW, Steven AS, Glenn H, editors. Radiometry: Jones and Bartlett Publishers, Inc. pp. 221-244.

4. Zhengyou Z. Flexible camera calibration by viewing a plane from unknown orientations; 1999 1999. pp. 666-673 vol.661.

5. Falcao G. HN, Massich J., and Fofi D. (2009) Projector-Camera Calibration Toolbox. Tech. Rep.
